# The use of digital photographs for the diagnosis of hand osteoarthritis: the AGES-Reykjavik study

**DOI:** 10.1186/1471-2474-13-20

**Published:** 2012-02-16

**Authors:** Helgi Jonsson, Gudrun P Helgadottir, Thor Aspelund, Johanna E Sverrisdottir, Gudny Eiriksdottir, Sigurdur Sigurdsson, Gudmundur J Eliasson, Asbjorn Jonsson, Thorvaldur Ingvarsson, Tamara B Harris, Lenore Launer, Vilmundur Gudnason

**Affiliations:** 1 Landspitalinn University Hospital, University of Iceland, IS-108 Fossvogur, Reykjavik, ICELAND; 2 University of Iceland, Reykjavik, ICELAND; 3 Icelandic Heart Association, University of Iceland, Reykjavik, ICELAND; 4 Icelandic Heart Association, Kopavogur, ICELAND; 5 Roentgen Domus Medica, Reykjavik, ICELAND; 6 Akureyri Central Hospital, Akureyri, ICELAND; 7 National Institute on Aging, Bethesda, MD, USA

**Keywords:** Hand ostearthritis, Generalized osteoarthritis, Epidemiology, Imaging, Photography

## Abstract

**Background:**

The objective of the study was to standardize a method using digital photographs to diagnose and grade hand osteoarthritis (HOA), to compare it with radiographs and clinical examination with regard to prevalence and relation to symptoms, and finally to construct a simple shortened version suitable for use in very large studies, where a global estimate may be preferable.

**Methods:**

High quality photographs with standard distance and hand positioning were analysed for the presence of HOA and subsequently compared with standard radiographs and clinical examination in 381 random participants in the AGES-Reykjavik Study, a large population study. The mean age of the participants was 76 years.

**Results:**

Using the photographic method, the most commonly affected joints were the second DIP joints followed by the third DIP joints and second and third PIP joints. Both interobserver (ICC = 0.83) and intraobserver reading agreements (ICC = 0.89) were acceptable. On comparison with radiography and clinical examination, aggregate scores were significantly correlated (R_s _0.35-0.69), more so in females (R_s _0.53-0.72) than males. Hand pain in males showed very little association with HOA findings by the three methods but all methods showed a comparable moderate association with hand pain in females. The performance of photography in predicting pain on most days for at least a month in females was comparable to that of radiography and clinical examination (AUC 0.63 *p *= 0.004). Analysis of intermittent pain yielded similar results for in the DIP and PIP joints (OR 3.2-3.3, *p *< 0.01), but for the CMC1 joints, both radiography (OR 9.0, *p *< 0.0001), and clinical examination (OR 9.8, *p *< 0.0001), had higher predictive odds ratios for pain than photography (OR 3.6, *p *< 0.0001)., A shortened, rapidly performed form of reading photographs also showed a high degree of correlation with the other methods (R_s _0.56-0.82).

**Conclusion:**

High quality hand photographs can be used to diagnose and grade hand osteoarthritis. The method has the advantage of being inexpensive and easy to perform. By using a slightly simplified method of reading, it appears to be highly suitable for use in large studies.

## Background

Hand osteoarthritis (HOA) is an important cause of pain and disability in the middle aged and elderly [1-3]. In addition to symptoms directly related to the hand itself, it is also related to osteoarthritis at other sites and there is evidence that the presence of HOA increases the propensity for the development and progression of both knee and hip osteoarthritis (OA) [4]. This relationship, usually referred to as "generalized osteoarthritis" (GOA) has proved hard to define, despite some serious efforts [5,6]. The reasons are complex and among them are gender and age related differences and problems with measures of the disease activity and progress. Imaging is particularly problematic, radiography has been considered the gold standard for diagnosis and monitoring of HOA, but the method is basically a delayed reflection of damage and repair caused by OA, showing only moderate association with symptoms and giving little information about prognosis [7,8]. Other imaging modalities such as scintigraphy, ultrasound and magnetic resonance imaging (MRI) have the advantage of being more dynamic and thus give a better indication of disease activity in the various tissues of the joints. All of these methods may be useful, but for various reasons including cost, availability and interpretation, none of them is likely to replace radiography in larger studies. The use of hand photographs as a screening method for HOA has been investigated in a few studies but found to be less sensitive than radiography [9-11].

The pathogenesis of osteoarthritis is still obscure and theories are evolving. Previously, OA was considered a degenerative disease, and simply an inevitable part of ageing. Now, however, OA is increasingly viewed as a dynamic process, one that is metabolically active, with the process of the disease involving both destruction and repair that may be triggered by a variety of biochemical as well as mechanical insults [12].

Members of our group have considerable experience in using different diagnostic methods for HOA including clinical examination [13], scintigraphy and MRI, both cross-sectional and longitudinal [14-16] and radiography with both the Kellgren-Lawrence and the Verbruggen-Veys scoring systems [17,18]. Different subsets have also been studied including hypermobile subjects and individuals with known genetic mutations [19,20]. Based on this experience, we think it may be time to consider HOA as trait or disease burden which is not well measured by any of the current methods and that larger studies involving investigations of other organ systems may be necessary for further understanding of the systemic factors involved in osteoarthritis. There is evidence that HOA is a systemic disorder interacting with other organ systems and sharing pathophysiological pathways with other conditions such as atherosclerosis [21,22]. This association was discovered only by linking photographic HOA information to the extensive database of information gathered in the AGES-Reykjavik Study [21].

Hand photography is a simple and inexpensive imaging method involving no ionizing radiation or discomfort. This project of using photographs to diagnose and grade HOA is based on the belief that by standardizing the taking and the reading of the photographs, we would acquire a method that would be very suitable for use in large studies, allowing a wide variety of association studies. This article describes the process of establishing this method in four main steps: (1) To develop a standardized reproducible grading system for the diagnosis of hand osteoarthritis from high quality hand photographs. (2) To compare the photographic scoring system with the two main diagnostic methods currently used; radiography and clinical examination. (3) To analyze the relationship between the three methods and hand pain, the main symptom of hand osteoarthritis (4). To construct a shortened version of the photographic method as a diagnostic tool suitable for very large samples, where a simple global assessment of HOA may be preferable.

## Methods

The Age, Gene/Environment Susceptibility-Reykjavik Study (AGES-Reykjavik) study is a population based study of approximately 5700 elderly individuals from the 40 year long Reykjavik study. They were aged between 66 and 96 and randomly recruited between 2002 and 2005. Details of the investigations are described in the study's baseline article [23]. The participants had extensive laboratory and imaging investigations including high quality hand photographs.

### Hand osteoarthritis study sample

Preliminary power analysis based on the expected prevalence of hand OA indicated that a sample of approximately 400 individuals would be suitable for comparison of photography and radiography for the diagnosis of hand OA. Between the months of February and June of 2005, 389 random AGES-Reykjavik Study subjects agreed to participate. Participation involved having a detailed clinical examination of the hands and a hand radiograph taken. Other diseases affecting visual assessment or the development of hand OA were recorded (e.g. inflammatory arthropathies, Dupuytren's contracture, neuropathies, post-traumatic) and those subjects disqualified. Thus there were 381 eligible participants, 159 males and 222 females.

### Hand pain documentation

Participants were questioned about hand symptoms with the following questions: 1) Have you ever had pain lasting at least one month in the joints of your hands or wrist? (The ACR criterion for diagnosis of hand OA). 2) In the past 12 months have you had pain lasting at least one month in the joints of your hands? 3a) Do you sometimes have pain in the joints of your hands? 3b) If yes, participants were asked to fill out a diagram showing in which joint the pain was located.

### Radiographic procedure

Standard radiographs were taken of both hands. All radiographs were examined by two experienced radiologists (GJE and AJ). Interreliability was found to be excellent (ICC = 0.87). Consensus scores were reached at a second sitting. The degree of radiographic OA in individual joints was graded using the Kellgren-Lawrence scoring system [17] (0 = absence; 1 = doubtful; 2 = mild; 3 = moderate; 4 = severe). Grade 2 or higher was considered a definite sign of radiographic OA.

### Clinical hand examination

All subjects were examined by an experienced clinical examiner (HJ). Individual hand joints were scored on the basis of structural changes, i.e. bony enlargement and deformity but not pain, on a 0-3 scale as follows: 0 = no evidence of OA, 1 = suspected but not definite OA, 2 = definite moderate OA, 3 = severe OA. Grade 2 or higher was considered a definite sign of clinically diagnosed OA.

### Photographic reading procedure

All photographs were taken with a Fuji Finepix 6800 zoom camera with images taken at 2800 × 2200 pixels. The camera was mounted on a tripod with a fixed distance to a black velvet board with markers for thumb positioning. The quality of the digital images and correct thumb positioning is important in order for the readers to be able to visually assess the degree of enlargement and deformity.

A photographic scoring system was developed. Initially, the readers (HJ, GPH) examined a few photographs at a time recording a number of variables that were suspected to be related to hand osteoarthritis in each joint. Each individual hand joint was graded separately. Subsequently the observers results were compared with each other and with hand radiographs. After a number of sessions, the variables most likely to be associated with clinical and radiographic hand OA were determined. Several factors were found to be of importance, such as hard tissue enlargement, visible soft tissue swelling, position and deformity.

The distal interphalangeal (DIP) and the proximal interphalangeal (PIP) joints were scored on a 0-3 scale as follows: 0 = no evidence of OA, 1 = suspected but not definite OA, 2 = definite moderate OA, 3 = severe OA.

For the DIP joints, the deformity of a joint without hard tissue enlargement did not justify the diagnosis of hand OA on its own but when deformity was severe (> 30°), the recorded score was raised by one (1) unit (to the maximum score of 3).

**Figure 1:**
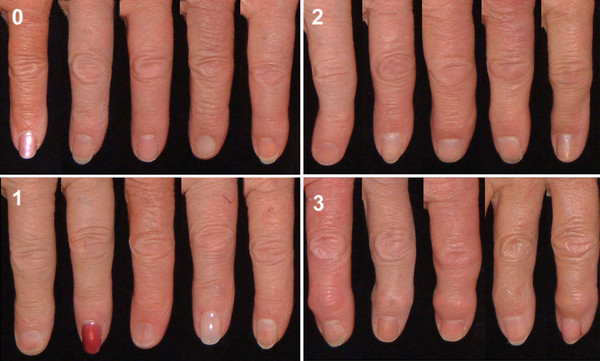
**Reference photographs showing the grading of osteoarthritis of the right second DIP joint**. The joint is given a score (0-3) for hard tissue enlargement and deformity of the joint.

**Figure 2:**
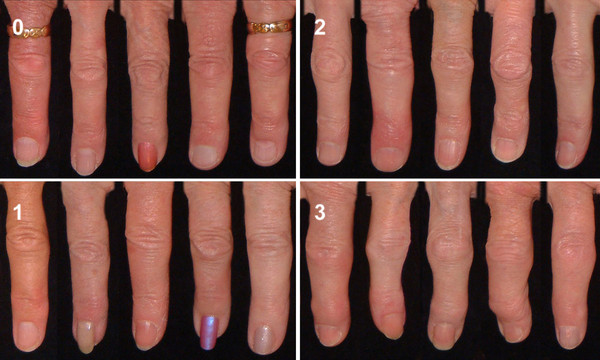
**Reference photographs showing the grading of osteoarthritis of the right third PIP joint**. The joint is given a score (0-3) for hard tissue enlargement and deformity of the joint.

Reference photographs for the grading of DIP and PIP joints are shown in Figures 1 and 2. For uniformity of presentation the right second DIP and third PIP joints are shown.

For assessment of OA of the first carpometacarpal (CMC1) joints, a slightly different approach was needed. Two different findings, enlargement of the joint and abnormal positioning, were related to OA in that joint. Abnormal positioning reflects palmar migration of the base of the first metacarpal bone and is reflected on photography by a number of factors, including disappearance of the normal configuration of the CMC1 joint, medial rotation of the thumb showing increased folding of the skin over the first metacarpal joint (MCP1) and sometimes hyperextension of that joint.

**Figure 3:**
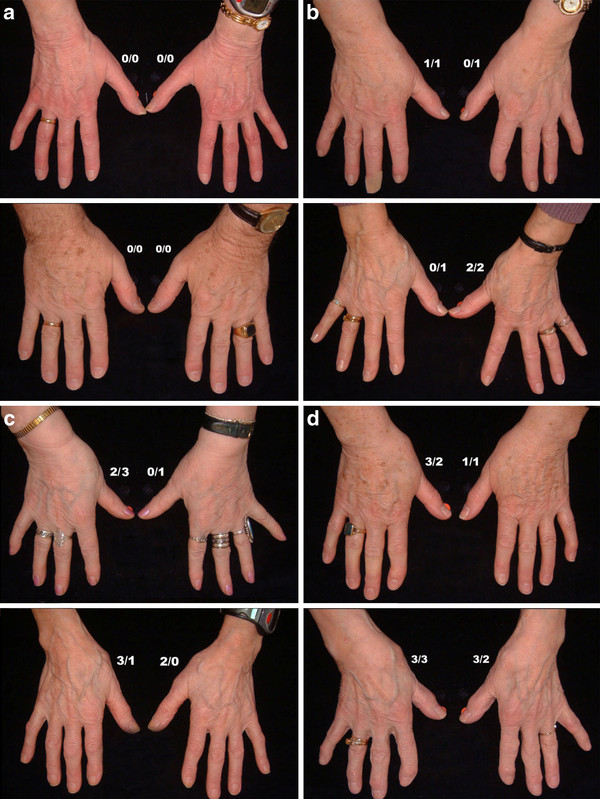
**Reference photographs showing the grading of osteoarthritis of the CMC1 joints**. The number on the left is the score for enlargement of the joint (0-3) and the number on the right represents position/subluxation of the thumb (0-3). a) Healthy CMC1 joints. b, c, d) Increasing osteoarthritis of the CMC1 joints.

Both enlargement and position were scored on a 0-3 scale, (0 = no evidence of OA, 1 = suspected but not definite OA, 2 = definite moderate OA, 3 = severe OA.) and subsequently added, giving a score of 0-6 which was translated into a 0-3 score as follows: (0 = Normal joint, 1 = Doubtful OA, 2-3 = Definite OA and 4 + = Severe OA). Reference photos for the CMC1 joints are shown in Figure 3.

The reference photographs were subsequently used as assistive tools in the reading of all photographs. Interreader agreement measured by ICC was good (average 0.83) and intrareader agreement (50 photographs re-read at 4 weeks intervals, GPH) was excellent (ICC 0.89) (Table 1). Finally the readers re-examined all discordant readings and decided upon a consensus score.

**Table 1:** Inter- and intraobserver agreement for photograph reading measured by Kappa and Average Measure Intraclass Coefficient (ICC)

	Joint	Interobserver	Interobserver	Intraobserver
		kappa	ICC	ICC
**Left**	DIP5	0.83	0.85	0.90
	DIP4	0.87	0.83	0.91
	DIP3	0.85	0.84	0.93
	DIP2	0.80	0.84	0.95
	PIP5	0.92	0.79	0.81
	PIP4	0.94	0.78	0.90
	PIP3	0.86	0.86	0.92
	PIP2	0.84	0.81	0.81
	CMC1	0.87	0.88	0.91
**Right**	DIP5	0.82	0.84	0.87
	DIP4	0.88	0.80	0.94
	DIP3	0.88	0.85	0.93
	DIP2	0.79	0.78	0.95
	PIP5	0.95	0.83	0.88
	PIP4	0.97	0.80	0.89
	PIP3	0.89	0.87	0.89
	PIP2	0.84	0.81	0.84
	CMC1	0.89	0.89	0.95
Average for all 18 joints		0.87	0.83	0.89

### Statistics

All statistical analyses were carried out with SPSS (v. 16.0) and SAS/STAT (version 9.2). For estimates of interobserver and intraobserver reliability and agreement for assessment of individual joints Kappa (on/off) (where grade 2 was used as cut-off point) and Average Measure Intraclass Correlation Coefficient (ICC) were used.

In order to compare severity measures of OA between the three methods photography, radiography and clinical examination we used an aggregate score from 10 joints (The second and third DIP joints, second and third PIP joints and the CMC1 joint on either side). The same score was also used to investigate the relationship with reported pain

Due to prevalence differences between the genders, prevalence data were calculated for males and females separately. Spearman rank correlation coefficient (R_s_) was used to assess correlations. The associations between reported pain and diagnosis of osteoarthritis by photographs, clinical examination, and radiography were compared with a logistic regression model. We assessed the odds ratios (ORs) with 95% confidence intervals for the DIP, PIP, and CMC1 joint groups separately, adjusting for age, BMI, smokingstatus and education level.

A receiver operating characteristic (ROC) curve analysis was performed for accuracy of the three methods in predicting pain.

## Results

The baseline characteristics of the study participants are shown in Table 2. The joint for joint prevalence of photographic osteoarthritis in the interphalangeal joints is shown in Tables 3, 4 and 5. Distal interphalangeal joint (DIP) OA was more common than proximal interphalangeal joint (PIP) OA in both genders and definite and severe OA was more prevalent in females. There was a slight right side predominance of OA. The most prevalent DIP joint was the right DIP2 joint and for the PIP joints it was the right PIP3 joint.

**Table 2:** Baseline characteristics of the study participants

	All participants(n = 381)	Males(n = 159)	Females(n = 222)
Age (SD)	76(5.0)	76(4.4)	76(5.3)
Heigth(SD)	167.2(9.2)	175.6(6.4)	161.2(5.5)
Weight(SD)	76.6(14.0)	83.7(13.0)	71.5(12.4)
Body Mass Index (BMI)	27.4(4.3)	27.1(3.9)	27.5(4.6)
Hand joint pain 1 month ever (ACR criterion)%	20.2	10.7	27.0
Hand pain lasting 1 month past year%	13.1	4.4	19.4
Hand pain sometimes%	28.3	10.1	41.4

**Table 3:** Consensus photographic scores for the DIP and PIP joints for all subjects

		Grade				
			
	Joint	0	1	2	3	
		*n*	*n*	*n*	*n*	*Total n*
**Right hand**						
	DIP5	163	143	66	7	379
	DIP4	231	109	38	2	380
	DIP3	201	113	50	12	376
	DIP2	99	154	100	22	375
	PIP5	281	67	30	0	378
	PIP4	320	47	11	2	380
	PIP3	214	103	54	8	379
	PIP2	192	140	43	2	377
**Left hand**						
	DIP5	180	137	55	7	379
	DIP4	232	118	23	4	377
	DIP3	183	136	53	6	378
	DIP2	116	169	85	10	380
	PIP5	303	52	26	0	381
	PIP4	349	22	5	3	379
	PIP3	255	91	29	5	380
	PIP2	213	144	21	2	380

**Table 4:** Consensus photographic score for the DIP and PIP joints for males

		Grade				
			
	Joint	0	1	2	3	
		*n*	*n*	*n*	*n*	*Total n*
**Right hand**						
	DIP5	73	59	25	1	158
	DIP4	103	42	13	0	158
	DIP3	91	45	17	2	155
	DIP2	43	78	37	0	158
	PIP5	116	30	11	0	157
	PIP4	129	22	7	0	158
	PIP3	81	46	28	2	157
	PIP2	72	60	24	0	156
**Left hand**						
	DIP5	73	61	24	0	158
	DIP4	99	49	7	0	155
	DIP3	83	57	16	0	156
	DIP2	43	78	37	0	158
	PIP5	121	21	17	0	159
	PIP4	145	11	1	0	157
	PIP3	96	43	17	2	158
	PIP2	83	64	11	0	158

**Table 5:** Consensus photographic score for the DIP and PIP joints for females

		Grade				
			
	Joint	0	1	2	3	
		*n*	*n*	*n*	*n*	*Total n*
**Right hand**						
	DIP5	90	84	41	6	221
	DIP4	128	67	25	2	222
	DIP3	110	68	33	10	221
	DIP2	46	92	65	17	220
	PIP5	165	37	19	0	221
	PIP4	191	25	4	2	222
	PIP3	133	57	26	6	222
	PIP2	120	80	19	2	221
**Left hand**						
	DIP5	107	76	31	7	221
	DIP4	133	69	16	4	222
	DIP3	100	79	37	6	222
	DIP2	73	91	48	10	222
	PIP5	182	31	9	0	222
	PIP4	204	11	4	3	222
	PIP3	159	48	12	3	222
	PIP2	130	80	10	2	222

The prevalence of OA in the CMC1 joints is shown in Table 6. For this joint we found more distinct gender differences. Severe OA was only seen in females and the prevalence of definite or severe OA in the right CMC1 join in females was 21.6% vs 5.7% in males. There was also a tendency towards more involvement on the right side.

**Table 6:** Consensus photographic scores for the CMC1 joints

		Grade				
			
	Joint	0	1	2	3	
		*n*	*N*	*n*	*n*	*Total n*
**All**	**CMC1 Right**	261	62	46	11	380
	**CMC1 Left**	290	50	29	9	378
**Males**						
	**CMC1 Right**	127	22	9	0	158
	**CMC1 Left**	141	12	4	0	157
**Females**						
	**CMC1 Right**	134	40	37	11	222
	**CMC1 Left**	149	38	25	9	221

### Comparison of photography with radiography and clinical examination

According to the photographic method, 60.4% of males had at least one affected hand joint, 85.5% had radiographic OA and 74.2% clinically diagnosed OA in at least one of the 18 hand joints. In females, the percentages were 66.2%, 93.7% and 82.4%, respectively.

We searched for confounding factors affecting the prevalence of HOA by the three methods. Despite a slight trend for increasing prevalence of HOA with age, we found no significant association with age for any of the methods. Weight (and BMI), however showed a significant negative association with HOA scores by the photographic method (*p *< 0.001) but not with the other two methods suggesting that the prevalence of HOA in overweight subjects may be underestimated on photographic assessment. Other possible confounding factors, such as smoking history and education were not associated with HOA scores by any of the methods.

The prevalences of Hand OA in the DIP, PIP and CMC1 joints by the three different methods is shown in Table 7. The cut-off points were chosen as those determining "definite OA" in at least one joint. It is evident that the sensitivities of the methods vary, with radiography being the most sensitive of the three for all joint groups. Compared with the other two methods, radiography is notable for a higher prevalence of OA in the PIP joints compared with the CMC1 joints and relatively minor gender differences. The other two methods are more similar with a relatively high prevalence of CMC1 HOA in females (comparable to or more prevalent than PIP OA) and low prevalence in males.

**Table 7:** Point prevalence of Hand OA in joint groups by the three methods.

	Males (N = 159)			Females (N = 222)		
	
	Total	Right	Left	Total	Right	Left
POA of DIPs%	48.4	39.0	32.7	50.0	45.0	34.7
POA of PIPs%	36.5	28.3	20.1	27.5	22.5	12.2
POA of CMC1%	5.7	5.7	2.5	25.2	21.7	15.4
ROA of DIPs%	81.8	78.0	66.7	91.9	88.3	80.6
ROA of PIPs%	50.3	35.8	32.1	67.6	50.0	43.2
ROA of CMC1%	25.8	18.2	19.5	35.1	27.9	28.4
COA of DIPs%	69.2	61.0	53.5	75.7	68.9	59.0
COA of PIPs%	28.9	20.8	18.9	22.5	18.5	14.9
COA of CMC1%	13.2	7.5	10.1	36.0	26.1	26.6

(POA = photography, ROA = radiography, COA = clinical examination)

**Figure 4:**
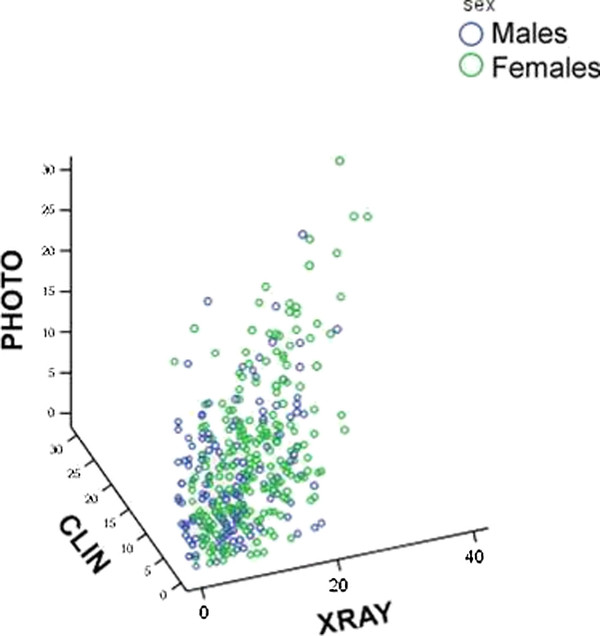
**A three dimensinonal scatter plot showing the total scores for the 10 ACR reference joints for each method, Photographic (PHOTO), Radiographs (XRAY) and clinical examination (CLIN)**.

All scoring methods showed significant correlations, somewhat stronger in females than in males. To give an indication of a score reflecting degree of involvement, we chose to present a 10 joint aggregate score, choosing the joints that are used in ACR diagnostic criteria (DIP2, DIP3, PIP2, PIP3 and CMC1 on both sides. They are referred to as P10 for the photographic scoring and R10 and C10 for radiography and clinical examination respectively). In males the Spearman correlation (R_s_) between P10 and R10 was 0.35 (ICC 0.55), for P10 and C10 0.69 (ICC 0.82) and R10 and C10 0.47 (ICC 0.72). Corresponding correlations for females were between P10 and R10: 0.53 (ICC 0.75), for P10 and C10: 0.72 (ICC 0.88) and R10 and C10: 0.66 (ICC 0.83). A three dimensional scatter plot for aggregate 10 joint scores for the three methods is shown in Figure 4.

### The relationship between the three diagnostic methods and pain

**Figure 5:**
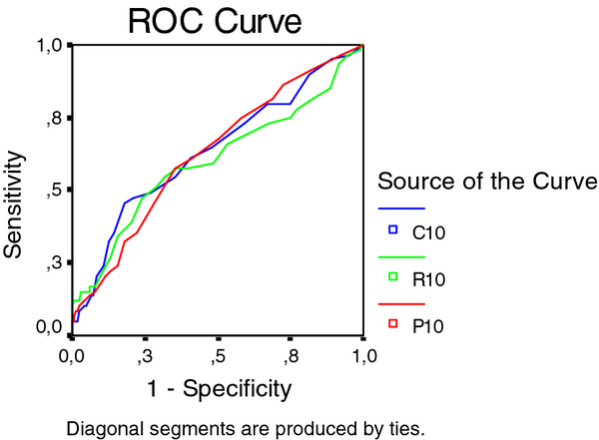
**A receiver operating characteristic (ROC) for "having hand pain lasting at least one month" (the ACR criterion for hand OA) in relation to aggregate scores of the 10 ACR reference joints the three diagnostic methods in females**.

Initially, we started out with three different pain criteria but the question "Hand pain lasting at least one month in the past year" had a low prevalence of positives (Table 1) and we did not find associations with any of the diagnostic methods. The prevalence of ever having hand pain lasting at least one month (the ACR criterion for diagnosis of hand OA) was 20.0% (10.7% in males and 27.0% in females) (Table 1). A positive answer in males was not associated with HOA by any of the methods, but in females there was a modest association with aggregate HOA scores by all three methods (Figure 5).

**Figure 6:**
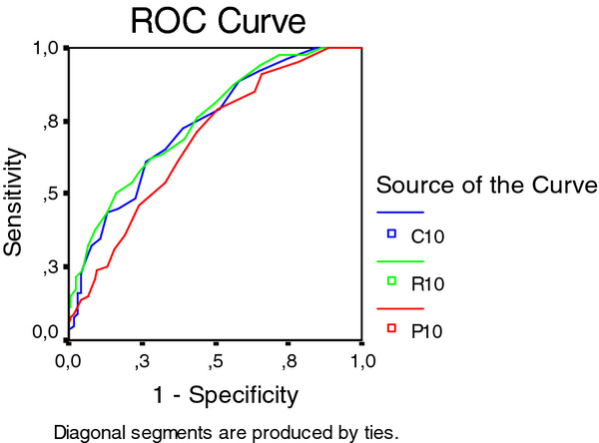
**A receiver operating characteristic (ROC) for "having hand pain sometimes" in relation to aggregate scores of the 10 ACR reference joints the three diagnostic methods in females**.

Sixteen males (10%) and 92 females (41.4%) reported "pain sometimes". In males there was no association between OA in DIP and PIP joints and either of the pain criteria. There was however, a significant association between intermittent pain in the CMC1 joints and HOA severity measured by radiography (OR 7.4 (1.2-46.4), *p *< 0.01 and clinical examination 14.3 (1.8-112.4), *p *< 0.001). The number of individuals in this group was low. In females, intermittent pain in individual joints and joint rows was significantly associated with the severity of OA assessed by all three methods (Figure 6).

Further analysis of the associations between intermittent pain in individual joints in females is illustrated in Table 8, showing odds ratios for the most commonly affected joint in each joint group.

**Table 8:** Odds ratios for reporting intermittent pain in relation to the presence of osteoarthritis by the three different methods.

	Percentage reporting pain having OA in the joint	Percentage reporting pain without having OA in the joint	Odds ratios and 95% CI	*P *value
**Right DIP2**				
Photo	29.3	11.6	3.2 (1.6-6.4)	0.001
Xray	21.5	4.4	5.9 (1.4-25.4)	0.008
Clinical examination	24.0	10.2	2.8 (1.2-6.2)	0.01

**Right PIP3**				
Photo	34.4	13.7	3.3 (1.4-7.6)	0.004
Xray	27.9	11.7	2.9 (1.4-6.0)	0.003
Clinical examination	35.7	14.1	3.4 (1.4-8.1)	0.004

**Right CMC1**				
Photo	41.7	16.7	3.6 (1.8-7.2)	< 0.0001
Xray	51.6	10.6	9.0 (4.4-18.2)	< 0.0001
Clinical examination	53.4	10.5	9.8 (4.8-20.1)	< 0.0001

Calculations for the most commonly affected joints in each joint group are shown. (right DIP2, right PIP3 and right CMC1)

### The shortened version of photographic assessment of hand osteoarthritis

**Figure 7:**
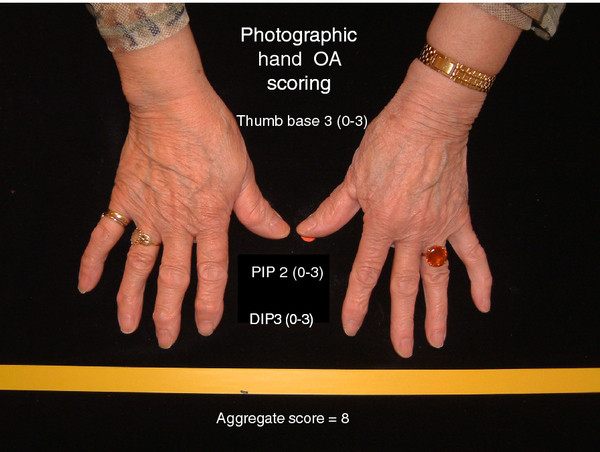
**An example of the use of the abbreviated photographic scoring system for hand OA**. Aggregate scores of ≥ 4 were assigned a score of 4, the most severe grading (HOASCORE).

**Figure 8:**
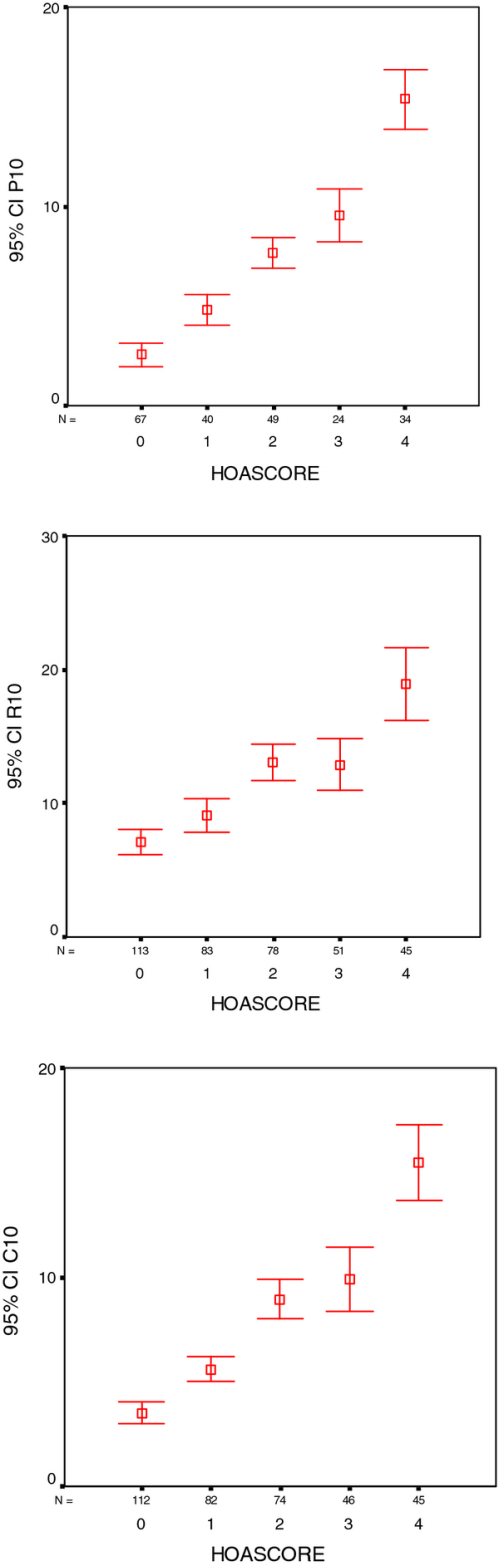
**Error bars (mean, 95% confidence interval) showing the relation between the three diagnostic methods and the shortened version of the photographic assessment (HOASCORE)**. P10 (Photographic), R10 (Radiographic) and C10 (clinical examination) stand for aggregate scores for the 10 ACR reference joints.

In view of the projected use of the photographic method as a screening tool for HOA, particularly in large studies, we constructed a shorter version of the scoring system for practical reasons. In the shorter version (HOASCORE) less attention was paid to the number of affected joints but more on a global assessment in an attempt to describe a trait or disease burden. Thus, the emphasis was on severity in each joint group (DIP, PIP, CMC1) with additional considerations for symmetry and typical joints (ACR 10). Thus, definite nodal OA (score 2 or higher on the reference photographs) on one side, or bilateral suspected OA (scores of 1) were classified as 1 (some evidence of HOA). Bilateral definite nodal OA was required for a score of 2 (definite HOA) and bilateral definite OA plus one or more severely affected joints were required for a global score of 3 (severe HOA) at each site. Adding the scores for the three joint groups resulted in an aggregate score of 0-9, subsequently truncated to zero to four or more (4+). An example of the use of the shortened version is shown in Figure 7. The scores obtained with the shortened version were highly correlated with the joint for joint scores for photography (P10) (R_s _0.82) and also had reasonably good correlations with radiography (R10) (R_s _0.56) and clinical examination (C10) (R_s _0.70). This is further illustrated in Figure 8.

This shortened version was subsequently applied for scoring the whole AGES-Reykjavik cohort (n = 5170). The prevalences of photographic HOA by this method (results for the AGES Reykjavik cohort in parestheses) were 0 (No HOA) = 30.3% (31.2), 1(some evidence of HOA) = 23.1% (22.6), 2 (definite mild HOA) = 20.7% (19.7), 3 (definite moderate HOA) = 13.8% (13.5) and 4+ (severe HOA) = 12.0% (13.0).

## Discussion

This paper describes the use of high quality digital hand photographs for the diagnosis and severity grading of hand osteoarthritis. The study population was a random sample of 381 elderly (mean age 76) participants in the AGES-Reykjavik Study who also had standard hand radiographs and expert clinical hand examination in addition to the digital photographs.

In the first step of the study, we developed a set of reference photographs to facilitate the reading and grading of the hand photographs. These were chosen after repeated assessments. By the help of these reference photographs, we managed to achieve an interobserver agreement measured by ICC which is comparable to that reported in radiological studies [24-26].

In the second phase of the study, we compared hand photographs with the results of radiography and clinical examination in the same group of individuals with regard to the diagnosis of HOA, and the grading of HOA severity. Unfortunately, the definition of HOA is very problematic because of lack of an absolute clinical, radiological, or pathological standard that the epidemiology of hand OA can be compared with. The ACR criteria for clinical diagnosis of HOA [27] are useful for identifying HOA patients with persistent symptoms but the prevalence of HOA by those criteria is low. A study of an elderly population in Iceland based on the ACR criteria [13] found that the prevalence of symptomatic hand OA was 3% in men and 7% in women. The symptoms criterion, however, showed considerable variation with time and thus the symptomatic OA group was not stable.

Radiological changes are most commonly used to grade hand OA. At present, several different radiographic classification systems are used but the Kellgren-Lawrence (K-L) scale for grading of radiological changes has been most widely used in the past [17]. In a review by Marshall and colleagues in 2008 it was reported that in 1996-2005 thirty epidemiological studies, all using the K-L scale, used 13 different cut-off points for diagnosis of systemic HOA [28]. The prevalence of symptomatic HOA is also low using the radiographic criteria, in the Framingham Study it had a point prevalence of 1.8-5.5% [29].

In the present study, we found several differences between the three methods. Radiography was more sensitive than either of the other methods when the cut-off was set at K-L ≥ 2. Radiography also had a higher relative prevalence in the PIP joints than the other methods. Aggregate scores for all methods showed highly significant correlations and with few exceptions, they tended to identify the same individuals as having severe HOA. Not unexpectedly, photographs and clinical examination results were more closely correlated to each other than to radiography. There were a few individuals with high radiography scores and low photographic and clinical scores (non-nodal hand osteoarthritis), which may constitute a relevant subset of HOA and will be the subject of further studies. Agreement between the three methods was considerably better in females than males. Weight and BMI were negatively associated with photographic HOA scores but despite reports indicating a positive association between radiographic HOA and weight we found no such association [30]. The photographic finding is not entirely unexpected and probably related to increased finger soft tissues hindering the detection of nodes and deformities.

Pain is the central symptom of OA and in the next phase of the study, we investigated the relationship between pain and HOA detected by the three methods.

Somewhat surprisingly, we found no association between HOA and pain in males except in those with CMC1 OA who admitted to pain in that joint. This could be related to the high age of our participants but some previous studies have reported a weaker association between radiographic HOA and pain in males than females [31,32]. In females, we found only a modest similar association between "pain in hands lasting at least one month" and aggregate severity scores for all three methods. The prevalence of this symptom and the weak association are in analogy with that found in previous studies [7]. Intermittent pain (pain sometimes) was more strongly related to HOA findings, both aggregate HOA scores and pain in individual joints. All three methods performed similarly in the case of the DIP joints, but radiography showed a somewhat stronger association with pain than photography for PIP and CMC1 joints. For the PIP joints, radiography also appeared superior to clinical examination.

In the final step of the study plan, we developed a shortened version of the photographic system. If information about HOA severity could be collected from some of the large detailed studies like the AGES-Reykjavik Study, it would open up a number of possibilities to examine the relationship of HOA to lifestyle and all kinds of conditions. If we consider HOA severity as a continous trait, the exact prevalences are less important than information the relative burden of HOA in each individual.

Using photographs for diagnosing HOA has a number of advantages. The method is simple, inexpensive, and involves little discomfort and no radiation. This study shows that the taking and the reading of the photographs can be standardized in a reproducible fashion with adequate inter- and intraobserver variation, at least in this age group. The photographic method in this age group is also in many ways comparable to the other methods, identifying mainly the same patients and showing comparable or only slightly inferior association with symptoms. Compared with clinical examination it also has the advantage of having an image for later analysis. The shortened version (HOASCORE) has practical advantages, speeding up the reading and appears to be particularly suitable for analysis of very large studies such as the AGES-Reykjavik Study where information about HOA status can be analysed in relation to the extensive health-related information available on each participant. By applying the HOASCORE to the AGES-Reykjavik Study population, we have discovered a number of potentially important new systemic associations, undetectable except in large studies [4,21,33].

The disadvantages of photography is that it is less sensitive on joint for joint analysis than either radiography and clinical examination particularly on PIP and CMC1 analysis. Also, compared with radiography it reflects anatomy less well. Bone damage or repair cannot be evaluated and the method cannot be used to diagnose erosive OA. The photographic scores also negatively associated with individual weight suggesting lower sensitivity in heavy subjects. Finally, a recent study suggests that photographic scoring of HOA is relatively insensitive to change, at least in this age group [34].

Of course, our conclusions regarding the use of photographs to diagnose HOA are limited to the current age group. In many ways this is a suitable age group to examine since it reflects cumulative disease burden and organ damage aquired over a long time. There is no reason to believe that photography performs differently in other age groups. In the future, it is even possible that photographs will prove to be more sensitive than radiographs in younger subjects with early disease who have nodal HOA but have not had time to develop radiological changes.

## Conclusions

High quality hand photographs can be used to diagnose and grade hand osteoarthritis in the elderly. The method has the advantage of being inexpensive and easy to perform. By using a slightly simplified method of reading, it appears to be highly suitable for use in large studies.

## Abbreviations

ACR: American College of Rheumatology; BMI: Body Mass Index; MRI: Magnetic Resonance Imaging; CMC1: First carpometacarpal (thumb base) joint C10: Clinical hand osteoarthritis severity score for 10 joints (DIP2: DIP3: PIP2:PIP3 and CMC1 on both sides; DIP: Distal interphalangeal joint; HOA: Hand osteoarthritis; ICC: Average Measure Intraclass correlation coefficient; K-L: Kellgren-Lawrence scoring system; OA: Osteoarthritis; P10: Photographic hand osteoarthritis severity score for 10 joints (DIP2: DIP3: PIP2: PIP3 and CMC1 on both sides; PIP: Proximal interphalangeal joint; R10: Radiographic hand osteoarthritis severity score for 10 joints (DIP2: DIP3: PIP2: PIP3 and CMC1 on both sides; GOA: Generalized osteoarthritis; GPH: Guðrún P. Helgadóttir; HJ: Helgi Jonsson.

## Competing interests

The authors declare that they have no competing interests.

## Authors' contributions

HJ, Main author. GPH, Data acquisition, Manuscript preparation. TA, Statistical analysis, Manuscript preparation. JES, Data acquisition, Critical reading of manuscript. GE, Project management, Critical reading of manuscript. SS, Data acquisition, Critical reading of manuscript. GJE, Data acquisition, Critical reading of manuscript. AJ, Data acquisition, Critical reading of manuscript. TI, Data acquisition, Critical reading of manuscript. TBH, Project management, Manuscript preparation. LL, Project management, Critical reading of manuscript. VG, Project management, Manuscript preparation. All authors read and approved the final manuscript.

## Pre-publication history

The pre-publication history for this paper can be accessed here:

http://www.biomedcentral.com/1471-2474/13/20/prepub
